# Editorial: Training and testing in climbing

**DOI:** 10.3389/fspor.2022.1006035

**Published:** 2022-08-17

**Authors:** Vidar Andersen, Jiří Baláš, Michail Lubomirov Michailov, Atle Hole Saeterbakken

**Affiliations:** ^1^Faculty of Education, Arts and Sports, Western Norway University of Applied Sciences, Sogndal, Norway; ^2^Faculty of Physical Education and Sport, Charles University, Prague, Czechia; ^3^Department Theory and Methodology of Sports Training, National Sports Academy, Sofia, Bulgaria

**Keywords:** performance, injury, bouldering, lead climbing, speed climbing, athletes, climbers

Climbing is often introduced as a naturally activity in childhood and to some extent follow us throughout the lifespan. For some climbing becomes more time consuming either as a hobby or a sport. Climbing has grown in popularity in the recent decades (Stien et al., 2022) and the popularity will probably continue to grow as climbing has been enrolled in the Olympics Games. Importantly, climbing is a relatively young area of research, and the evidence-based knowledge is limited. Performance in climbing is affected by multiple physiological, psychological, and technical factors (Vigouroux and Quaine, 2006; Baláš et al., 2012; Philippe et al., 2012). To develop reliable tests, improve performance, and avoid injuries in climbing, several gaps of knowledge need to be filled. Therefore, the Research Topic “*Training and testing in climbing*” aimed to increase the scientific knowledge of climbing related to testing and training.

Forty-eight authors originating from Europe and North America have contributed to the 13 manuscripts being published in this Research Topic. The study design includes one mini-review, eight cross-sectional studies, two randomized control trials (RCT), one longitudinal follow-up, and one case study. These studies provide new knowledge to different fields (i.e., training methodology, physiology, psychology, and medicine). Thus, the Research Topic's interdisciplinary evidence has several applications: (a) increasing the informativeness of tests for assessing and monitoring climbing-specific fitness; (b) optimization climbing-specific training; (c) development of new training methods based on the physiological functions as well as motor and mental abilities that most strongly determine climbing performance.

In sport climbing, there are three different disciplines (Woollings et al., 2015). Winkler et al. conducted a cross-sectional study to determine the external load's volume characteristics in the different disciplines at an international level. Video recordings from the 2018 World Cup and the 2018 World Championships showed great variations between the disciplines according to number of moves, time per move and the ratio between activity and rest.

To be able to assess performance related factors, valid and reliable tests are essential (Baechle and Earle, 2008). Three of the studies presented in this Research Topic focus on testing procedures. The mini-review of Stien et al. provided an overview of the climbing-specific tests, procedures and outcomes used to assess climbing performance and training effects. Twenty-five studies were included, and the tests were categorized into climbing-specific endurance-, strength, and power tests. The review showed a disagreement between protocols, and multiple approaches to assess climbing-related strength, power and endurance. Importantly, few studies have reported the reliability and validity of their tests. Regarding testing, Augste et al. conducted a cross-sectional study aiming to find procedures for an intermittent finger flexor endurance test to optimize the correlation with lead climbing performance. The authors concluded that the highest correlations were found for women when 9% deviation in the required force and 1 second deviation in the pulling time was tolerated. For men, the optimum was reached with the same time deviation and a force deviation of 6%. Maciejczyk et al. also evaluate distinct performance indicators in addition to energy system contributions in four different finger flexor tests: maximal finger strength, a 30-s all-out, a continuous, and an intermittent endurance. The authors concluded that maximal grip force and all-out isometric contractions are equally decisive indices of climbing performance. Further, maximal grip force reflects maximal anaerobic power, while all-out average force and force time integral of constant isometric contraction at 60% of maximal force are functional measures of anaerobic capacity. Aerobic energy demands for the intermittent exercise are dominated by the aerobic re-phosphorylation of high-energy phosphates.

Finger flexor strength has been considered as one of the most important physiological factors for climbing performance (Saul et al., 2019). Three of the included studies in the Research Topic focused on finger flexor strength. In a cross-sectional study, Vereide et al. investigated the difference of maximal force and rate of force development (RFD) in male sport climbers. Seventy-eight climbers performed an isometric pull-up on a rung. The authors concluded that maximal force and RFD are greater among climbers on higher performance levels. Further, there is a moderate-to-strong association between maximal and rapid force production and climbing performance. Both Hermans et al. and Devise et al. conducted RCTs aiming to evaluate the effect of hangboard training. Hermans et al. concluded that among intermediate to advanced climbers, 10 weeks of hangboard training increases the maximal finger strength to larger extent than regular climbing training. Devise et al. reached a similar conclusion when they conducted a 4-week intervention among advanced to elite climbers comparing regular climbing to training at an intensity of 60, 80, or 100% of MVC. Of the three intensities, 80% of MVC was the only intensity improving both maximal strength, endurance and stamina. In prolongation of intensity in climbing, Baláš et al. examined the possibility of applying the mathematical model of critical power to the estimation of a critical angle as a measure of maximal metabolic steady state (critical angle) in climbing. Twenty-seven climbers at an intermediate to advanced level conducted multiple ascents at different angles on a treadwall. The authors concluded that a predefined route with three to five different wall angles may be used to estimate critical angle as an analog of critical power. Moreover, using muscle oxygen breakpoint determined by near-infrared spectroscopy from a climbing test with progressive increases in wall angle also appears to provide a valid estimate of critical angle.

Two studies focused on other factors that may affect training and performance. Limmer et al. examined the use of compression garments. Compression garment has received scientific interest in the recent years (Brown et al., 2017) and may improve sport performance (Yang et al., 2020). Limmer et al. compared the immediate effects between forearm compression sleeves, non-compressive placebo forearm sleeves, or no forearm sleeves on sports climbing performance. Based on the results it was concluded that forearm compression has no effect on climbing performance. Marcen-Cinca et al. compared the visual perception system in climbers at different levels through a psychophysical optical test. The findings indicated that elite to high elite climbers performed better at the visual perception tasks compared to the advanced climbers. There were no differences between the groups in the visual acuity and contrast sensitivity tests.

Two studies focused on some of the less positive sides of training and elite sports. Joubert et al. aimed to determine the prevalence of amenorrhea among elite level competitive sport climbers. An online survey was distributed with a response rate of 114 female sport climbers. A total of 18 athletes were presented with current amenorrhea in addition to 14 athletes provided information that indicated irregular cycles. Pastor et al. focused on the longitudinal effects of climbing conducting a 10-year follow-up study where they aimed to investigate the 10-year changes in cortical bone thickness, base osteophyte occurrence and radiological signs of osteoarthritis in the fingers of male sport climbers. The results showed that climbing at the elite level likely induces mechano-adaptation of cortical bones in the fingers, and build-up takes place over the career. Further, climbers show higher frequencies of base osteophytes compared to none-climbers. Radiographic signs of osteoarthritis seem to increase throughout the climbing career. Regarding rehabilitation, Vagy conducted a case study on evaluating and treating a female climber with posterior elbow pain using Telehealth. Exercises were reviewed during the initial evaluation with video and verbal feedback to confirm correct exercise performance. After 10 weeks, the climber's pain decreased from 4/10 to 0/10. Further, she made a full recovery back to her previous grade and was able to perform at the same level as before the injury.

As this Research Topic is finalized, we, the guest editors, believe it has strengthened the evidence-based knowledge concerning training and testing in climbing. Further, we hope that it has brought valuable and practical information which coaches, athletes and recreational climbers can benefit from. Finally, as the research-area of climbing is still in an early stage, we hope the included studies can motivate other researchers around the world to start new projects, bringing the evidence-based knowledge of climbing forward.

## Author contributions

VA, JB, MM, and AS wrote and edited the manuscript. All authors contributed to the article and approved the submitted version.

## Conflict of interest

Authors MM and JB are currently affiliated with Climbro, a private company who provides hangboards with integrated force sensors and mobile application for climbing specific training. The remaining authors declare that the research was conducted in the absence of any commercial or financial relationships that could be construed as a potential conflict of interest.

## Publisher's note

All claims expressed in this article are solely those of the authors and do not necessarily represent those of their affiliated organizations, or those of the publisher, the editors and the reviewers. Any product that may be evaluated in this article, or claim that may be made by its manufacturer, is not guaranteed or endorsed by the publisher.
